# 600. The Effect of Medication-Assisted Treatment on Completion Rates of Outpatient Parenteral Antibiotic Therapy

**DOI:** 10.1093/ofid/ofab466.798

**Published:** 2021-12-04

**Authors:** Christian S Greco, Mohammad Mahdee Sobhanie, Kelci E Coe, Courtney Hebert, Margaret Williams

**Affiliations:** 1 The Ohio State Wexner Medical Center, Columbus, OH; 2 The Ohio State University Wexner Medical Center, Columbus, OH; 3 Ohio State University Wexner Medical Center, Columbus, OH; 4 The Ohio State University College of Medicine, Columbus, OH

## Abstract

**Background:**

Injection drug use is a nationwide epidemic associated with an increased risk of invasive Staphylococcus aureus (S. aureus) infections. Medication-assisted treatment (MAT) is effective in reducing substance use and increasing adherence to inpatient medical therapy in persons with injection drug use (PWID). Studies assessing the impact MAT has on completion of outpatient parenteral antibiotic therapy (OPAT) are limited.

**Methods:**

This was a single-center, retrospective, cohort study at The Ohio State University Wexner Medical Center in patients admitted from 12/1/2017 to 12/1/2019 with a diagnosis of S. aureus bacteremia who were identified as PWID either by ICD-9 or 10 code or chart review. A formal MAT program was established on 11/30/2018. Patients were assigned to the pre-MAT group if they were discharged prior to 11/30/2018 and to the MAT group with treatment after 11/30/2018. We evaluated a composite outcome of failure to complete OPAT, recurrence of S. aureus bacteremia during the OPAT period and readmission within 30 days. A multivariable logistic regression analysis was performed to examine the association between MAT therapy and the primary composite outcome, while adjusting for proven confounders.

**Results:**

A total of 700 patients were identified with 644 patients omitted based on exclusion criteria. The study population included 27 in the Pre-MAT group and 17 in the MAT. Median age was 37 years (IQR 30.6 - 46.1). There was a higher number of females in the MAT therapy group compared to the pre-MAT group (82% vs. 33%, p=0.002). Patients in the pre-MAT group had a significantly longer length of stay (25 days vs. 17 days, p=0.01). The primary composite outcome was met if a patient did not complete their OPAT, if they had a recurrence of S. aureus bacteremia during their OPAT or if they were readmitted to the hospital within 30 days. In the pre-MAT group 14/27 (52%) met the composite outcome versus 6/17 (35%) of the MAT group (p=0.28).

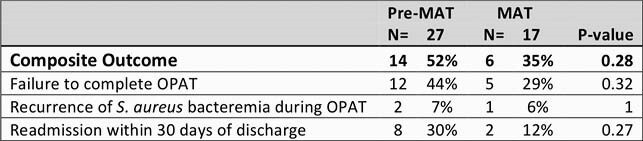

**Conclusion:**

Patients in the MAT group met the composite outcome 17% less than those in the pre-Mat group which is suggestive of the impact MAT has on completion of OPAT therapy; however, this study did not reach statistical significance as it was underpowered. Further longitudinal evaluation with greater sample size is needed to fully evaluate this intervention.

**Disclosures:**

**Mohammad Mahdee Sobhanie, M.D.**, **Regeneron** (Scientific Research Study Investigator)**Regeneron** (Scientific Research Study Investigator, Was a sub-investigator for Regeneron 2066 and 2069)

